# Dissolvable Polyvinyl-Alcohol Film, a Time-Barrier to Modulate Sample Flow in a 3D-Printed Holder for Capillary Flow Paper Diagnostics

**DOI:** 10.3390/ma12030343

**Published:** 2019-01-22

**Authors:** Dorin Harpaz, Tim Axelrod, Alicia Lu Yitian, Evgeni Eltzov, Robert S. Marks, Alfred I.Y. Tok

**Affiliations:** 1 School of Materials Science & Engineering, Nanyang Technological University, Singapore 639798, Singapore; DORIN001@e.ntu.edu.sg (D.H.); ALU003@e.ntu.edu.sg (A.L.Y.); 2 Department of Biotechnology Engineering, Ben-Gurion University of the Negev, Beer-Sheva 84105, Israel; timaxel@post.bgu.ac.il; 3 Institute for Sports Research (ISR), Nanyang Technological University and Loughborough University, Nanyang Avenue, Singapore 639798, Singapore; 4 Agriculture Research Organization (ARO), Volcani Centre, Rishon LeTsiyon 15159, Israel; eltzov@volcani.agri.gov.il; 5 The National Institute for Biotechnology in the Negev, Ben-Gurion University of the Negev, Beer-Sheva 84105, Israel; 6 The Ilse Katz Centre for Meso and Nanoscale Science and Technology, Ben-Gurion University of the Negev, Beer-Sheva 84105, Israel

**Keywords:** polyvinyl alcohol, dissolvable membrane, time barrier film, paper based sensors, sample flow control, 3D-printed holder

## Abstract

Integrating a dissolvable membrane into a sensor allows the control of sample flow, location and duration in critical areas. These time-barrier films stop the flow of samples until the membrane has dissolved, thus, for example, allowing increased exposure time between immunoreagents for the formation of greater numbers of immuno-complexes, ensuring higher sensitivity, reactivity, and helping to reduce false-positive signals. In this study, dissolvable polyvinyl alcohol (PVA) films are used in a 3D-printed sensor holder, which enables film integration without the use of glue. PVA is a synthetic hydrophilic linear polymer, its solubility is dependent on its molecular weight and degree of hydrolysis. Three types of PVAs films were tested herein: (1) PVA 1-Mw: 30–70 K, 87–90% hydrolyzed; (2) PVA 2-Mw: 31–50 K, 98–99% hydrolyzed and (3) PVA 3-Mw: 89–98 K, >99% hydrolyzed. The films were exposed to water in (1) the novel 3D-printed holder and (2) directly immersed into a water droplet. After comparing the time taken to dissolve PVA 1–3 films, PVA 1 films of 5–20% (*w/v*) are found to be most suitable as time barrier films, due to their optimal dissolution times and physical properties for integration into the customized 3D-printed holder.

## 1. Introduction

A dissolvable membrane can be integrated into a sensor in order to allow control of sample flow. The membrane acts as a time-barrier film, by stopping the sample flow until it is dissolved. The control of sample flow in sensor critical areas, such as conjugation and blocking layers, allows increased exposure time between immunoreagents for the formation of more immuno-complexes. This helps to ensure higher sensitivity, reactivity and in some cases reduces false-positive signals [1,2,3,4]. The ideal material to function as a time-barrier film should be stable in dry form but still dissolves easily upon wetting, thus allow the flow of the sample. A recent study demonstrated the use of a polyacrylamide-based dissolvable membrane in a microfluidic chemical and biological sensor. In the presence of the sample, the membrane dissolves and permits the sample flow into interdigitated electrodes. This results in a resistance change between the electrodes and transduces the chemical event into an electrical signal [5]. However, in order for such dissolvable membranes to be employed in high-speed sensing technologies, the total operation time of the device should be approximately 30 min or less. This should include the time taken for the time-barrier films to dissolve, enable optimal protein-antibody reaction, and signal testing. As a result, the time taken for the time-barrier films to dissolve should ideally be within the range of 10–15 min [6].

Dissolvable films contain a water-soluble polymer, such as hydroxylpropyl-methylcellulose, polyvinylpyrrolidone, polyvinyl-alcohol, carboxymethyl-cellulose, polyethylene-oxide, hydroxylpropyl-cellulose, hydroxylethyl-cellulose, methyl-cellulose, pullulan, gelatin, pectin, sodium alginate, maltodextrin, polymerized rosin, and xanthan [7]. The polymer of choice is usually selected based on its brittleness as well as its rate of dissolution, which is inversely related to its molecular weight. Moreover, plasticizers, such as glycerol, propylene glycol, poly (ethylene glycol), glycerine, dimethyl phthalate, diacetyl phthalate, dibutyl phthalate, triacetrin, castor oil, citrate ether, and tryethyle citrate are added for the control of the film mechanical properties [8]. In addition, there are two main methods for dissolvable film preparation: Solvent-casting and hot-melt extrusion. In the solvent-casting method, the polymer is dissolved together with the plasticizer in a selected solvent (usually water or ethanol), which is later spread out using casting and drying technique. This method is ideal for heat-sensitive materials, however can contain trace amounts of the solvent used. In the hot-melt extrusion method, the dry form of the ingredients are heated and extruded until they are mixed. Then, the melted material is forced through an extrusion die to form the desired shape. In this method there is no addition of solvent material, however the material can go through thermal degradation due to high temperature used [9,10].

In this study, a PVA film was employed inside capillary flow paper diagnostics, in order to control the sample flow. Polyvinyl alcohol (PVA) is a synthetic hydrophilic linear polymer which forms copolymers of vinyl alcohol and vinyl acetate. PVA was first synthesized by Hermann and Haehnel in 1924 by hydrolyzing polyvinyl acetate in ethanol with potassium hydroxide [11]. Since vinyl alcohol is unstable and rapidly tautomerizes into acetaldehyde, PVA is commercially produced via the hydrolysis of Polyvinyl Acetate. As such, the physical and structural properties of PVA are dependent on the extent of polymerization and hydrolysis. Esterification, etherification, crystallization, ion-polymer complexation in the polymer can be caused by changes in these factors [12]. The unique properties of these modified PVA structures, such as their adhesiveness, strength, film forming, biocompatibility, swelling, safety, and non-carcinogenicity, are beneficial in different industries including textile, paper, adhesives, food, biomedical, and pharmaceutical in particular [13].

PVA is widely used as a non-protein blocking agent in immunoassays to saturate the unoccupied sites on the solid phase. This prevents non-specific binding of the immune-reactants, since it does not get contaminated by interfering proteins like immunoglobulins or endogenous peroxidases [14,15,16,17,18]. Moreover, PVA is widely used as a humidity-sensing material. The hydrophilic functional groups in PVA give the polymer its high affinity to water. They absorb the moisture in the air, which changes the water content in the PVA film [19,20,21,22]. In addition, since PVA is a water soluble polymer, it is also widely used in the creation of biodegradable packaging. Plasticizers, such as glycerol, are added for increased flexibility and reduced brittleness, whereas surfactants, such as tween 20, are added to enhance the plasticizer function [23]. Together with reagents such as chitosan, cellulose, and cross linking reagents, a biodegradable film which is highly dissolvable in water is created [24,25,26,27,28,29,30].

PVA is highly soluble in water as it comprises a large amount of hydroxyl groups, which interact with the water molecules through hydrogen bonds. The PVA solubility depends on its degree of hydrolysis and molecular weight [31]. The degree of hydrolysis of the PVA is inversely proportional to its solubility. PVA with a high degree of hydrolysis will be less soluble in water due to inter- and intra- molecular H-bonds that form between the hydroxyl groups of the PVA molecules. With higher degree of hydrolysis, there will be greater interactions amongst the PVA molecules than with the water molecules, thus PVA solubility is reduced. Conversely, with a lower degree of hydrolysis, inter- and intra- molecular H-bonds are reduced due to steric hindrance from a large quantity of hydrophobic acetate groups. This helps to increase interactions between PVA molecules and water molecules, thus boosting solubility. Similarly, PVA with lower molecular weights will dissolve faster in water over higher molecular weights as they exhibit lesser intramolecular interaction [32]. Therefore, in this study, three types of PVAs films were tested herein: (1) PVA 1-Mw: 30–70 K, 87–90% hydrolyzed; (2) PVA 2-Mw: 31–50 K, 98–99% hydrolyzed and (3) PVA 3-Mw: 89–98 K, >99% hydrolyzed.

In this study, the films were integrated inside a 3D-printed holder, for the elimination of the use of glue, and the time taken for the dissolution of the films was measured. With the recent emergence of 3D printing technology, it has become possible to rapidly and easily produce customized objects. 3D printing has allowed for constant design-build-test cycles for objects ranging from microfluidic chips to smartphone interfaces, which are complements to sensor applications [33]. 3D-printing technology has allowed us the creation of a prototype that can securely hold the PVA film for it to function as a time barrier film inside a paper based sensor casing, without the use of glue. The ability of these time-barrier films was examined by employing them in the customized 3D-printed holder.

## 2. Materials and Methods

### 2.1. Materials

PVA 1, Mw: 30–70 K, 87–90% hydrolyzed (Cat. 8136), PVA 2, Mw: 31–50 K, 98–99% hydrolyzed (Cat. 363138), PVA 3, Mw: 89–98 K, >99% hydrolyzed (Cat.341584), Glycerol (Cat. G6279), and Tween20 (Cat. P7949) were purchased from Sigma-Aldrich. Sample (Cat. GFB-R4), absorbent (Cat. AP-080) and conjugate release matrix (Cat. PT-R5) pads were purchased from Advanced Microdevices Pvt. Ltd (20-21, Industrial Area, Ambala Cantt, Haryana, India). Nitrocellulose membrane 0.45 μm (Cat. No. 1620115) was purchased from Bio-Rad. Fullcure 810 VeroClear (OBJ-03271) and Resin Support Fullcure 705 (OBJ-03200) were purchased from Stratasys (Eden Prairie, Minnesota, United States). Parafilm “M” was purchased from Bemis (Neenah, WI, USA).

### 2.2. Equipment

Balance was purchased from Mettler Toledo. The hotplate (WH220PLUS) and the Binder oven were purchased from Gaia Science Pte Ltd. Film cutter (PAT/RE38, 219 & D613, 795) was purchased from EK Tools, Vaessen Creative (Vaessen B.V. Thermiekstraat 25 6361HB Nuth, The Netherlands). Digital Vernier Caliper was purchased from Kincrome. PowerBlast (high pressure water cleaner, 20-120 bar, manufactured by Balco UK for Objet Geometries Limited) and Inkjet 3D Printer (Stratasys Eden 260) were purchased from Stratasys (Eden Prairie, MN, USA).

### 2.3. Preparation of Films

Using the solvent-casting technique, the PVA films were made from aqueous polymer solutions. The PVA was first weighed, and then added into deionized water according to the required concentration. For the preparation of 1% PVA, add 0.1 g PVA slowly into 10 mL of deionized (DI) water. Once added, the solution was mixed on a hotplate at 250 rpm and 80 °C for 1 h to obtain a homogenous solution. The PVA solution was heated up to 80 °C in order to break up the crystal structure and thus allow it to dissolute. Then, 10 mL of the homogenous PVA solution was placed in a disposable plastic petri dish and placed into the oven at 37 °C for 22 h. The PVA solution volume was also varied and the following volumes were tested: 10 mL, 15mL, and 20 mL.

### 2.4. 3D-Printing of Customized Holder

The customized 3D-printed holder was modelled using Autodesk Fusion 360 software and printed using Stratasys Eden 260 Inkjet 3D Printer, using VeroClear and Sup705 as supporting material. Once printed, the prototypes were washed using PowerBlast to remove the excess support material.

### 2.5. Determination of Dissolution Time in Water

In order to eliminate factors such as, breaking or tearing of the film and poor exposure of film to sample within the 3D-printed holder, the dissolution time of the tested PVA 1–3 films was first determined under standard conditions. Each tested PVA film was cut into small circles of 6 mm diameter then immersed into a 100 ul DI-water droplet on a parafilm. The films were visually and physically examined to determine the time for dissolution.

### 2.6. Determination of Dissolution Time in the 3D-Printed Holder

**Figure 1:**
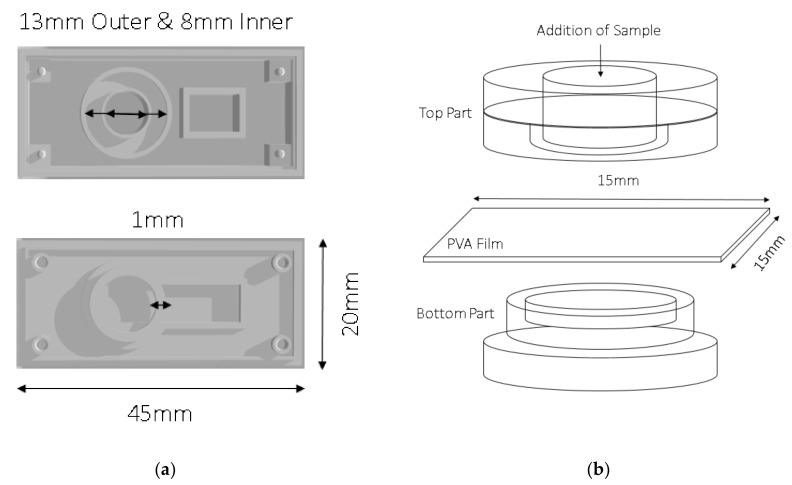
Schematic description of customized 3D-printed polyvinyl alcohol (PVA) film holder. (**a**) Top part: Outer circle with 13 mm diameter and inner circle with 8 mm diameter. Bottom part: Ring thickness of 1 mm. (**b**) PVA film (15 mm × 15 mm) integration and sample addition.

**Figure 2:**
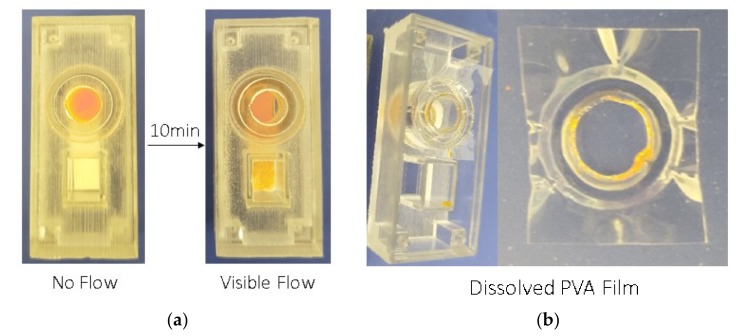
Control of sample flow. (**a**) Integration of a PVA film inside the customized 3D-printed holder, after applying ink sample, there is a visible delay of flow. It is clear to identify the sample flow after 10 min of waiting time. (**b**) Dissolved PVA film, after applying the ink sample and allowing for a waiting time of 30 min for full sample flow.

PVA 1–3 films were also integrated into customized 3D-printed holders and the time taken for the films to dissolve inside the holder was determined. First, an absorption pad was cut into 5mm x 20 mm and placed in the designated rectangle at the bottom of the holder. Then, each tested PVA film was cut into a 15 mm × 15 mm square which were placed between the top and bottom components of the 3D-printed holder, exactly on the ring of the bottom of the holder (Figure 1). After the tested PVA film was integrated into the holder an ink sample was then added through the top entrance of the holder. The dissolution time of each tested film was measured after observing the visible flow of the ink sample into the absorption pad (Figure 2). Since reagent-containing membranes are a common components of many sensors, the effect of such membranes on the dissolution time was also tested by adding sample pads above the PVA film before the addition of the ink sample.

### 2.7. Characterization of Film Weight and Thickness

The thickness of the tested PVA films was measured using a Digital Vernier Caliper. The weight of the tested PVA films was measured after the films were cut into 15 mm × 15 mm squares.

## 3. Results and Discussion

### 3.1. Comparison of PVA 1-3 Films Dissolving Time in Water

**Figure 3:**
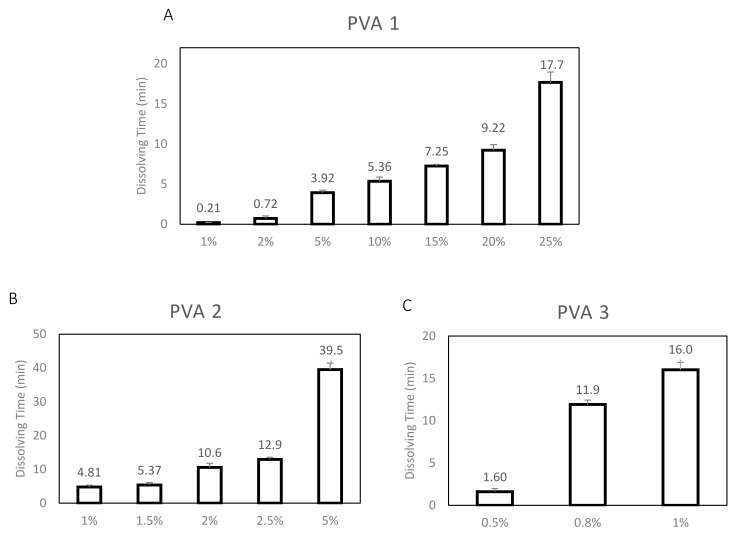
Comparison of PVAs 1–3 films dissolving time in DI-water. The dissolution time was determined after immersing the PVA films into 100ul of DI-water droplet on parafilm. Dissolution time was compared between three types of PVA: (**A**) PVA 1 (Mw: 30–70 K, 89–90% Hydrolysed); (**B**) PVA 2 (Mw: 31–50 K, 98–99% Hydrolysed) and (**C**) PVA 3 (Mw: 89–98 K, 99% Hydrolysed). Different concentrations were selected for each PVA type, according to the Mw and hydrolysis level.

Three types of PVA were tested for their use as time barrier films. The three types of PVA used were as follow: (1) PVA 1-Mw: 30–70 K, 87–90% hydrolyzed; (2) PVA 2-Mw: 31–50 K, 98–99% hydrolyzed and (3) PVA 3-Mw: 89–98 K, >99% hydrolyzed. The three types of PVAs were chosen based on the differences in their Mw and level of hydrolysis. Theoretically, PVA degree of hydrolysis is inversely proportional while the PVA Mw is directly proportional to its solubility [31,32]. As such, it was hypothesized that PVA 1 will dissolve most quickly because of its lower Mw and lower level of hydrolysis. This is followed by PVA 2, which has a higher level of hydrolysis, then PVA 3 due to its high Mw and high level of hydrolysis. The dissolution time of the tested PVA 1–3 films were first determined under standard conditions. Each tested PVA film was cut into small circles of 6mm diameter and immersed into 100 uL of DI-water droplet on a parafilm. The films were visually and physically examined to determine the time for dissolution and the results are presented in Figure 3. A range of PVA concentrations were used to obtain films with different physical properties for testing since their ease of integration into the 3D-printed holder is an essential factor. The dissolution time of the PVA 1–3 films was determined for the following concentrations: 1% to 25% (*w/v*), 1% to 5% and 0.5% –1% respectively. PVA 1 1% and 2% (*w/v*) films dissolved rapidly after less than 1min, while the PVA 1 25% (*w/v*) film dissolved too slowly, taking up to ~18min. These films were unsuitable as a time-barrier as they either did not provide sufficient time for the formation of the immuno-complexes or dissolved too slowly to be effective in a sensing device. PVA 1 5–20% (*w/v*) films, however, dissolved after 4–10 min, which is within the time range of interest for a time barrier film for paper based sensors. The PVA 2 films were tested with a narrower range of concentrations: 1% to 5% (*w/v*), as the films did not dissolve even after 2 h for 10% (*w/v*) concentration. The results presented in Figure 3B show that even a 5% (*w/v*) concentration gave a very long dissolution time of ~40 min. Although PVA 2 1–2.5% (*w/v*) films dissolved after 5–13 min, which is within the time range of interest, it was ultimately deemed unsuitable due to its brittleness which would be a problem during the film-holder integration The PVA 3 films dissolving time was determined in the concentrations: 0.5% to 1% (*w/v*), since 2%, 5% and 10% (*w/v*) films did not dissolve even after 2 h. The results presented in Figure 3C show that PVA 3 0.5% (*w/v*) films dissolved only after ~2 min while the PVA 3 0.8% and 1% (*w/v*) films dissolved after 12–16 min. Similarly, PVA 3 0.8% and 1% films were determined to be too brittle and thus unsuitable for use as a time-barrier film inside paper-based sensors. To conclude, after the comparison of PVA 1–3 films, PVA 1 5–20% (*w/v*) films were found to be the most suitable for use as time barrier films, due to their optimal dissolution times and physical properties for integration into the 3D-printed holder.

### 3.2. PVA 1 Film Dissolving Time in 3D-Printed Holder

**Figure 4:**
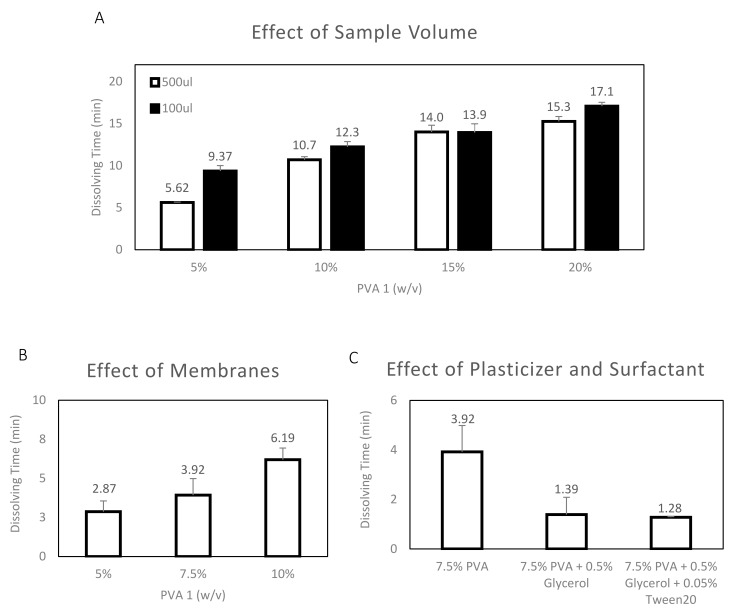
PVA 1 film dissolving time in customized 3D-Printed holder. (**A**) Effect of sample volume, comparing dissolution time with 100 uL and 500 uL of DI-water. (**B**) Effect of Membranes on 7.5% (*w/v*) PVA 1 film dissolution time, using 500 uL DI-water. (**C**) Effect of plasticizer and surfactant on 7.5% (*w/v*) PVA 1 film dissolution time, using 500 uL DI-water.

PVA 1 5–20% films were selected for further testing in the customized 3D-printed holder since they were determined to have optimal dissolution times and physical properties compared to the other tested films. After they were integrated into the holder, as described in Section 2.6, the dissolution time of each tested film was determined after the visible flow of the ink sample into the absorption pad (Figure 2) was observed. Moreover, the effect of three other factors, (1) sample volume, (2) presence of membranes, and (3) plasticizers and surfactants, on dissolution time were also tested and the results are presented in Figure 4. As presented in Figure 4A, the effect of sample volume on dissolution time was tested for two different volumes of sample: 100 uL and 500 uL. The resultant dissolution time is similar between the 100 uL and 500 uL samples: 10% (*w/v*): ~11–12 min; 15% (*w/v*): ~14 min and 20% (*w/v*): ~15–17 min, except for a small difference in the case of PVA 1 5% (*w/v*) films (100 uL: ~10 min, 500 uL: ~6 min). Moreover, the PVA 1 films have a longer dissolution time in the 3D-printed holder than in the water drop: 5% (*w/v*) - drop ~4 min vs. holder ~6 min; 10% (*w/v*) - drop ~5 min vs. holder ~11 min, 15% (*w/v*) - drop ~7 min vs. holder ~14 min and 20% (*w/v*) - drop ~9 min vs. holder ~15 min. The time taken for dissolution was longer in the 3D-printed holder as the exposure area of the PVA film to water in the 3D printed holder is limited to only a 6 mm Ø surface, whereas the PVA film was fully immersed inside the water droplet, which increased the exposure area. The results suggest that the 3D-printed holder can be employed in sensors to effectively tune the dissolution time of PVA films by adapting the area of film-sample exposure based on the needs of the sensor. As previously mentioned, reagent-containing membranes are a common component of many sensors. Therefore, the effect of the presence of membranes was also tested in the 3D-printed holder and the results are presented in Figure 4B. Five sample pads were placed on top of the PVA 1 films before the addition of the ink sample. The addition of membranes reduced the dissolution time of the PVA 1 films: 5% (*w/v*) without ~6 min vs. with ~3 min and 10% (*w/v*) without ~11 min vs. with ~6 min. Furthermore, the effect of adding plasticizer (0.5% (v/v) Glycerol) and surfactant (0.05% (v/v) Tween20) to the PVA film was also tested in the 3D-printed holder and the results are presented in Figure 4C. The plasticizer and surfactant effect was added to the PVA film with the intention of improving the film flexibility for better integration into the 3d-printed holder. However, from the resultant dissolution time, the plasticizer and surfactant addition into the PVA 1 film content reduced the dissolution time significantly from ~4 min to ~1 min.

### 3.3. PVA 1 Film Weight and Thickness Characterization

**Figure 5:**
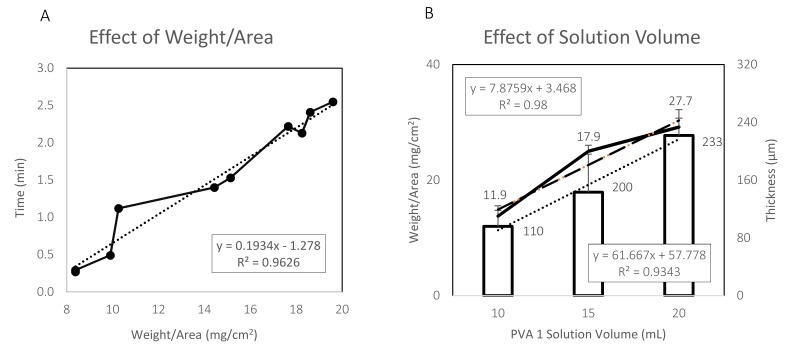
PVA 1 film weight and thickness characterization. (**A**) Effect of weight of 7.5% (*w/v*) PVA 1 film on dissolution time when immersed in 100ul of DI-water droplet on parafilm. (**B**) Weight and thickness as a function of PVA 1 solution volume.

The characterization of the weight and thickness of the PVA 1 films was also conducted. Results are presented in Figure 5. PVA 1 7.5% (*w/v*) films were prepared and then cut into 6mm diameter circles. The PVA circles were first weighed then tested for their dissolution time when immersed in a DI-water droplet on parafilm. From the results presented in Figure 5A, a linear connection between the PVA circles’ weight to its dissolution time was observed. After standardization per area, the linear connection between the dissolution time (min) to weight/area (mg/cm^2^) can be described with the following equation: y = 0.1934x−1.278, R^2^ = 0.9626. Moreover, the characterization of the weight and thickness of the PVA 1 7.5% (*w/v*) films based on three different PVA solution volumes was also conducted and the results are presented in Figure 5B. After the preparation of the PVA 1 7.5% (*w/v*) films, the films were cut into 15mm x 15mm squares. From the results presented in Figure 5B, a linear connection between the films’ weight to the solution volume can be observed. After standardization per area, the linear connection between weight/area (mg/cm^2^) to solution volume (ml) can be described with the following equation: y = 7.8759x + 3.468, R^2^ = 0.98. The linear connection between thickness (um) to solution volume (ml) can be described with the following equation: y = 61.667x + 57.778, R^2^ = 0.9343.

## 4. Conclusions

In this study, dissolvable PVA films are used in a novel, customized 3D-printed sensor holder for the control of sample flow. Integrating a dissolvable membrane inside a paper based sensor will allow the control of the sample flow in selected critical areas. With the use of PVA as a time barrier film, the sample will not flow until the required time. This allows increased exposure time between protein and antibody in order to allow more immuno-complexes to be formed in order to ensure higher sensitivity, reactivity, and reduced false-positive signal [1,2,5]. In addition, as previously discussed, the PVA solubility depends on its molecular weight and degree of hydrolysis. Three types of PVAs films were tested: (1) PVA 1-Mw: 30–70 K, 87–90% hydrolyzed; (2) PVA 2-Mw: 31–50 K, 98–99% hydrolyzed and (3) PVA 3-Mw: 89–98 K, >99% hydrolyzed. PVA 1 5–20% (*w/v*) films were found to be the most suitable for use as time barrier films, due to ideal dissolution times and physical properties for integration into the customized 3D-printed holder. As expected, the dissolution times of PVA 1 films were slower in the 3D-printed holder then in the DI-water droplet: 5% (*w/v*) drop ~4 min holder ~6min; 10% (*w/v*) drop ~5 min holder ~12 min, 15% (*w/v*) drop ~7 min holder ~14 min and 20% (*w/v*) - drop ~9 min vs. holder ~15 min. Moreover, a linear relation can be observed between the PVA weight/area and it’s dissolving time. As previously discussed, the intended use of the dissolvable PVA films is inside a paper-based sensor. A potential sensor platform is Stack-pad [34,35]. Stack-pad is based on vertically stacked functional membranes. The sample is added from the bottom-up, and as each layer is wetted, the analyte is pushed upwards. Through the immunocomplex (analyte-antibody conjugated with horseradish peroxidase (HRP)) migration, a measurable signal is produced. In order to prevent a false positive result, a proprietary blocking layer membrane is added to stop unbounded antibodies from reaching the top membrane. Thus, only the analyte/antibody-HRP complex will generate a signal. For example, the dissolvable PVA film has the potential to improve the sensitivity and specificity of the stack-pad sensor if it is employed in order to allow increased exposure time of the tested sample in the blocking layer membrane. To conclude, the dissolvable PVA film has the potential to improve the sensitivity and specificity of such sensors by allowing increased exposure time in the sensor critical areas, which will potentially increase immunocomplex formation.
